# A 20-month longitudinal study to evaluate humoral and cellular immunity after COVID-19 vaccines

**DOI:** 10.1590/0074-02760240193

**Published:** 2025-07-18

**Authors:** Wlademir Braga Salgado Sobrinho, Bárbara Batista Salgado, Aguyda Rayany Cavalcante Barbosa, Vanessa Araújo Passos, Lisvane Paes Vieira, Lhorruama Dias do Nascimento, Jaila Dias Borges Lalwani, Pritesh Jaychand Lalwani, Paulo Afonso Nogueira

**Affiliations:** 1 Universidade Federal do Amazonas, Programa de Pós-Graduação em Imunologia Básica e Aplicada, Laboratório de Doenças Infecciosas e Imunologia, Manaus, AM, Brasil; 2 Fundação Oswaldo Cruz-Fiocruz, Instituto Leônidas e Maria Deane, Laboratório de Diagnóstico e Controle de Doenças Infecciosas na Amazônia, Manaus, AM, Brasil; 3 Universidade Federal do Amazonas, Faculdade de Ciências Farmacêuticas, Manaus, AM, Brasil

**Keywords:** COVID-19, vaccines, longevity, humoral immunity, cellular immunity

## Abstract

**BACKGROUND:**

The effectiveness of coronavirus disease 2019 (COVID-19) vaccines is well established; however, the long-term durability of vaccine-induced immunity remains to be fully elucidated.

**OBJECTIVES:**

This study longitudinally compared humoral and cellular immune responses in two groups: G1, who received two doses of Sinovac-CoronaVac, and G2, who received two doses of AstraZeneca-Oxford, both subsequently boosted with Pfizer.

**METHODS:**

Immune responses were assessed at five time points: P1 (prior to the second dose), P2 (90-180 days after the second dose), P3 (pre-booster, six-eight months post-second dose), P4 (90-180 days post-booster), and P5 (180-270 days post-booster). Anti-Spike severe acute respiratory syndrome coronavirus 2 (SARS-CoV-2) IgG levels were measured by enzyme-linked immunosorbent assay (ELISA), while IFNγ-producing cells in response to Spike peptides were quantified using enzyme-linked immune absorbent spot (ELISPOT). IgG subclasses were analysed in a subset of samples.

**RESULTS:**

Following the first dose, Sinovac-CoronaVac induced lower anti-Spike IgG levels than AstraZeneca-Oxford, though levels equalised after the second dose. After the Pfizer booster, anti-Spike IgG levels remained elevated for up to six months in both groups. IgG1 was predominant in both groups, with occasional expression of IgG2 and IgG4. The mean frequency of IFNγ-producing cells was lower in the Sinovac-CoronaVac group before and up to six months after the second dose, compared to AstraZeneca-Oxford. However, post-Pfizer booster, both means became comparable. Between 90-180 days post-booster, the Sinovac-CoronaVac + Pfizer group exhibited a statistically significant decline in IFNγ-producing cells relative to the AstraZeneca-Oxford + Pfizer group. By P5, over half of individuals in the Sinovac-CoronaVac + Pfizer group demonstrated no detectable cellular response.

**MAIN CONCLUSIONS:**

High antibody levels were maintained for up to six months following both homologous and heterologous vaccination. However, cellular immunity declined more markedly in the Sinovac-CoronaVac + Pfizer group, resulting in a higher proportion of non-responders. These findings underscore the importance of tailored booster strategies to sustain protective immunity against COVID-19.

Coronavirus disease 2019 (COVID-19) is an infectious disease that can lead to severe cases of pneumonia, known as severe acute respiratory syndrome (SARS), resulting from a dysregulated immune response and cytokine storm. These cases can rapidly progress to sepsis, organ failure, and death.^1^ ^,^ ^2^ ^,^ ^3^ Since the beginning of the pandemic, severe acute respiratory syndrome coronavirus 2 (SARS-CoV-2) has infected more than 770 million people and caused nearly 7 million deaths, with a mortality rate of approximately 1%.^4^ ^,^ ^5^ The global impact of the pandemic has been unprecedented, prompting the accelerated development of vaccines, with 10 approved by the World Health Organization (WHO) and 13 billion doses administered worldwide by June 2023.^6^ ^,^ ^7^

The approved COVID-19 vaccines utilise various platforms and demonstrate efficacy against severe disease and hospitalisation.^7^ ^,^ ^8^ Currently, the vaccine platforms for SARS-CoV-2 include classical modalities such as live attenuated or inactivated viruses, as well as next-generation vaccines based on the Spike (S) protein of SARS-CoV-2 in recombinant viral vectors, in addition to mRNA and recombinant protein vaccines.^9^ The mechanism of protection provided by COVID-19 vaccines is not yet fully understood; however, it is widely accepted that vaccination induces both cellular and humoral responses, with T-cell responses correlating with neutralising antibody titres, respectively.^7^ ^,^ ^10^

In Brazil, the Ministry of Health, through the Department of Immunisation and Communicable Diseases and the General Coordination of the National Immunisation Programme of the Health Surveillance Secretariat, presented a plan for the operationalisation of COVID-19 vaccination in the country.^11^ Under this plan, the Pfizer-BioNTech (BNT162b2), AstraZeneca-Oxford (ChAdOx1), Janssen-Cilag (Ad26.COV2.S), and Sinovac (Sinovac-CoronaVac) vaccines were approved by the National Health Surveillance Agency (ANVISA) and made available to the population.

Recently, we compared the immune response and breakthrough infections following vaccination with Sinovac-CoronaVac and Oxford-AstraZeneca.^12^ Of the 10 breakthrough infections observed in the study, nine occurred in individuals vaccinated with Sinovac-CoronaVac and one (1) with Oxford-AstraZeneca. The study revealed that the lower efficacy is attributable to the fact that Sinovac-CoronaVac generates inferior immune responses, particularly in individuals without prior infection, whereas those who had a previous infection followed by vaccination (hybrid immunity) exhibited stronger and more durable antibody responses.

The present study is a longitudinal and prospective analysis of individuals who participated in the DETECTCoV19 seroprevalence studies.^13^ ^,^ ^14^ One group received two doses of the Sinovac-CoronaVac vaccine (Sinovac Life Sciences) and a third dose of the BNT162b2 vaccine (BioNTech-Pfizer) six months later. Another group received two doses of the ChAdOx1nCoV-19 vaccine (AstraZeneca-Oxford) and a third dose of the BNT162b2 vaccine six months later. The objective is to compare the longevity of humoral and cellular responses to determine the need for, and frequency of, booster doses within the national COVID-19 vaccination programme.

## SUBJECTS AND METHODS

**Fig. 1:**
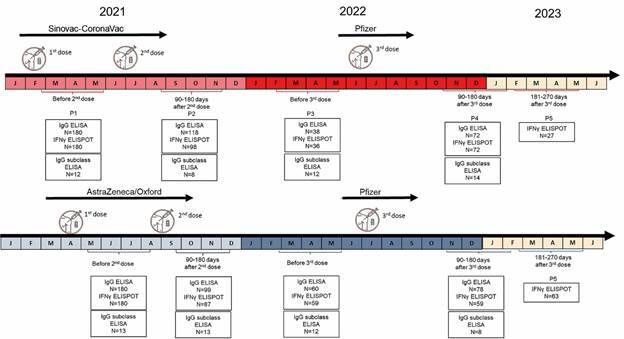
flowchart of the longitudinal assessment of immune response in participants of the DETECT-COVID survey following Sinovac-CoronaVac and AstraZeneca-Oxford vaccination. The study initially evaluated the humoral and cellular immune responses of 360 participants from the DETECT-COVID survey, with 180 in the Sinovac-CoronaVac arm (two doses of Sinovac-CoronaVac followed by one Pfizer booster) and 180 in the AstraZeneca-Oxford arm (two doses of AstraZeneca-Oxford followed by one Pfizer booster). Throughout the follow-up period, a substantial proportion of participants remained engaged in the study and returned for subsequent blood collections. Accordingly, the flowchart presents the exact number of participants and peripheral blood samples collected at each time point (P). It is important to note that, according to the national coronavirus disease 19 (COVID-19) vaccination schedule, the first two doses were administered during the first half of 2021, with the third dose offered at the end of 2021. In Manaus, the Sinovac-CoronaVac vaccine was introduced at the beginning of 2021, followed by the AstraZeneca-Oxford vaccine a few months later. In this study, antibody production was compared between the two groups, with reactivity assessed at four follow-up points: P1) before the 2nd dose; P2) between 90- and 180-days after the 2nd dose; P3) before the 3rd Pfizer dose (between 90- and 180-days after the 2nd dose); P4) between 90- and 180-days after the 3rd dose. To evaluate the longevity of cellular immunity, in addition to points P1 through P4, a fifth collection point (P5) was conducted between 181- and-270 days after the Pfizer booster dose.

*Type, location and population* - This is a cohort study with both retrospective and prospective components, with a follow-up period of up to one year. The study was conducted in collaboration with Dr Pritesh Lalwani’s laboratory at the Federal University of Amazonas. The Instituto Leônidas & Maria Deane (ILMD, Fiocruz-Amazonia) also participated in the study. The project entitled “DETECTCoV19” was conducted with more than 3,000 residents of Manaus, Amazonas.^13^ ^,^ ^15^ Participants for this cohort were recruited from this list, selecting individuals who were about to be vaccinated and agreed to participate in the study. A flowchart summarises the number of samples used (Fig. 1). The first arm consisted of participants who received the Sinovac-CoronaVac vaccine for both the first and second doses, followed by a third dose (booster) with the Pfizer vaccine; the second arm consisted of participants who received the AstraZeneca-Oxford vaccine for both the first and second doses, followed by a third dose with the Pfizer vaccine. At the first time point (P1), prior to the second dose (whether in the Sinovac-CoronaVac or AstraZeneca-Oxford arm), 180 participants were invited, and this number decreased at the second time point (P2), between 90 and 180 days after the second dose. Among participants in both arms, 118 vaccinated with Sinovac-CoronaVac returned, and 99 from the AstraZeneca-Oxford arm returned. The number of participants further decreased at the third time point (P3), prior to the third dose, which was the Pfizer vaccine. Only 38 of the initial 180 participants from the Sinovac-CoronaVac arm and 60 from the AstraZeneca-Oxford arm returned. At the fourth time point (P4), between 90 and 180 days after the third dose, the number of participants increased to 72 and 78, respectively. The fifth time point (P5) occurred more than six months after the third dose (between 181 and 270 days post-Pfizer), for the evaluation of the longevity of cellular immunity.

*SARS-CoV-2 recombinant Spike protein* - The Spike protein was kindly provided by Professor Dr Leda Castilho (Alberto Luiz Coimbra Institute for Graduate Studies and Research in Engineering - COPPE). Briefly, the HEK293-COV2-S cell line was stably transfected to express the soluble ectodomain of the SARS-CoV-2 Spike protein in its pre-fusion trimeric conformation. The cells were cultured in HEK-GM medium within bioreactors. Protein purification was carried out by affinity chromatography, as previously described in the referenced publication.^16^

*Serology by enzyme-linked immunosorbent assay (ELISA)* - Flat-bottom 96-well polystyrene microplates of the MaxiSorp^®^ type (Nunc, USA) were used as the reaction support. ELISA plates were coated with 100 ng of SARS-CoV-2 recombinant Spike protein in carbonate-bicarbonate buffer (pH 9.6) and incubated overnight at +4ºC. Plates were subsequently blocked with 10% skimmed milk in phosphate-buffered saline (PBS) at room temperature (20-25ºC), washed, and then incubated with patient plasma diluted 1:100 for 90 minutes.

For the detection of total IgG antibodies, goat anti-human IgG (SeraCare-KPL) was used. For the detection of IgG subclasses (IgG1, IgG2, IgG3, and IgG4), the following mouse monoclonal anti-human IgG antibodies (all from Sigma-Aldrich) were employed: anti-IgG1 (clone HP6001), anti-IgG2 (clone HP6014), anti-IgG3 (clone HP6050), and anti-IgG4 (clone HP6025), followed by a peroxidase-conjugated anti-mouse IgG (Sigma-Aldrich). All washing steps were performed four times using PBS-T buffer (0.05% Tween 20 in PBS). Blocking was carried out with 10% skimmed milk in PBS. Antibody incubation steps were conducted for 60 min at room temperature. For detection, a colourimetric reaction was developed by adding 50 μL of the light-sensitive substrate 3,3′,5,5′-tetramethylbenzidine (TMB). Levels of total IgG or IgG subclasses were expressed as optical density (OD).

To determine the reactivity index (RI), a panel of 20 serum samples from healthy individuals, collected before 2020 and stored in our sample bank, was used to establish a cut-off value for seropositivity. To do this, an ELISA was performed using 96-well plates coated with the Spike protein, and the OD values from these pre-pandemic samples were measured. The variation in OD values was used to calculate the cut-off for defining IgG anti-Spike positivity.

The cut-off was calculated using the formula: cut-off = mean OD of the 20 sera + [3 × standard deviation (SD) of OD]. A sample was considered positive for anti-Spike IgG if its OD value exceeded this cut-off. To quantify IgG levels, the RI was calculated as follows: RI = OD of the sample / cut-off value, indicating how many times higher the OD of the test sample was in comparison to the cut-off.

*Isolation of peripheral blood mononuclear cells (PBMCS)* - Whole blood was collected in heparinised tubes and centrifuged at 500 × g for 15 min to separate the cellular fraction from the plasma. The plasma was then removed and stored at -20 ºC. The cellular fraction was subsequently subjected to density gradient centrifugation using Ficoll-Paque (GE) to isolate PBMCs. Following washing, PBMCs were cryopreserved in cell recovery medium containing 10% dimethyl sulfoxide (DMSO; Gibco) and 90% heat-inactivated foetal bovine serum (FBS; Gibco), and stored in liquid nitrogen until use in downstream assays.

*Enzyme-linked immune absorbent spot (ELISPOT)-based assays for identifying IFN-gamma (IFNγ) secreting cells* - The assay quantifies effector T cells that respond to stimulation using two distinct peptide pools derived from the SARS-CoV-2 Spike protein (T-SPOT.COVID, Oxford Immunotec). Briefly, the T-SPOT.COVID antigen panel consists of overlapping peptides spanning the Spike protein sequence. The peptide design ensures maximal epitope coverage, comprising 253 peptides, and is intended to detect T-cell reactivity irrespective of HLA restriction, while avoiding cross-reactivity with other coronaviruses.

After collection, whole blood was diluted in RPMI 1640 medium at a 1:1 ratio, *i.e.*, 16 mL of blood mixed with 16 mL of RPMI, totalling 32 mL. The samples were homogenised, and Ficoll was added at a 1:2 ratio, *i.e.*, 16 mL of Ficoll to 32 mL of blood diluted in RPMI 1640 medium. The tubes were then centrifuged at 400 × g for 30 min at 25ºC. Following centrifugation, the PBMC layer (the buffy coat located between the plasma and Ficoll layers) was carefully collected using a Pasteur pipette. The cell suspension was transferred to a 15 mL Falcon tube, and the volume was adjusted to 15 mL by adding RPMI 1640 medium.

The samples were centrifuged again at 1,500 rpm for 7 min at 25ºC. Following centrifugation, the supernatant was discarded, and the pellet was resuspended in 15 mL of RPMI 1640 medium and centrifuged once more. After extraction, washing, and counting of PBMCs, the cell suspension was adjusted to a concentration of 2.5 × 10⁶ cells/mL in RPMI. A total of 2.5 × 10⁵ cells were added to each well. Stimulation conditions included Spike peptides (Oxford Immunotec), a positive control using phorbol 12-myristate 13-acetate (PMA), and a negative control containing culture medium only. Upon completion of the procedure, the plate was sealed and incubated at 37ºC with 5% CO₂ for 16 to 20 h.

*Plate revelation* - After overnight incubation, the wells were washed four times with 1X PBS. Subsequently, 50 μL of capture antibody solution (diluted 1:200) was added to each well and incubated for 1 h at 2-8ºC (refrigerated). Following incubation, the plate was washed again four times with 1X PBS. After the final wash, 50 μL of substrate solution - nitroblue tetrazolium/5-bromo-4-chloro-3-indolyl phosphate (NBT/BCIP) - supplied in the kit (Thermo Fisher) was added to each well in the dark. The plate was then covered with aluminium foil and incubated for 7 min. To stop the enzymatic reaction, the plate was washed with Milli-Q or distilled water at least twice. The wells were gently blotted dry on paper towels, and the plate was left to air dry overnight prior to spot enumeration.

*Spot counting* - Spots were automatically counted using the ELISpot Analyser - S6 UV (CTL immunospot, USA) at the Oswaldo Cruz Institute, Rio de Janeiro. A result was considered valid if the negative control well contained fewer than 10 spots and the positive control well contained 20 or more spots. If spots were detected in the negative control, this number was subtracted from the spot count in the test wells (COV-A and COV-B). For analysis purposes, the number of spots in the test wells was multiplied by four to obtain the count of spot-forming cells (SFC) per million PBMCs.

*Statistical analyses* - Data were tabulated in an Excel database created by the researchers and analysed using GraphPad Prism software, version 7. Descriptive statistics were presented as mean and SD for numerical variables with normal distribution, or as median and interquartile range (IQR) for non-normally distributed variables. For univariate analysis, continuous variables were compared between two groups using the Mann-Whitney U test. Statistical significance was denoted as follows: ^*^for p < 0.05; ^**^for p < 0.005; and ^***^for p < 0.0005.

*Institutional review board statement* - The study was conducted in accordance with the Declaration of Helsinki, and approved by the Research Ethics Committee of Federal University of Amazonas (UFAM) (CAAE no. 34906920.4.0000.5020).

*Informed consent statement* - Informed consent was obtained from all subjects involved in the study. Data Availability Statement: De-identified participant data can be made available to researchers after approval from the research ethics committee. The requests should be directed to the corresponding author (pritesh.lalwani@fiocruz.br). All participants provided both oral and written informed consent prior to enrolment. Regarding the eligibility criteria, individuals from the initial list who were scheduled to be vaccinated and agreed to participate in the cohort were selected. Inclusion criteria comprised pregnant women, postpartum women, and individuals with SARS-CoV-2 infection confirmed by serology, aged over 18 years. Exclusion criteria included individuals who had already received the first or second dose of any COVID-19 vaccine. Also excluded were individuals with insufficient data to complete at least 80% of the required fields in the data collection form, as well as those who did not complete the sample collection process according to the study protocol.

## RESULTS

*Comparison of anti-Spike IgG antibody levels in the two vaccine arms* - In the national vaccination schedule, the first two doses of the COVID-19 vaccine were made available during the first half of 2021, with the third dose introduced towards the end of that year (Fig. 1). In Manaus, the Sinovac-CoronaVac vaccine was made available at the beginning of 2021, while the AstraZeneca-Oxford vaccine was introduced a few months later. This study evaluated the humoral and cellular immune responses of 360 individuals who participated in the DETECTCoV survey.^13^ ^,^ ^15^ Of these, 180 were included in the Sinovac-CoronaVac arm (two doses of Sinovac-CoronaVac followed by one Pfizer booster), and 180 in the AstraZeneca-Oxford arm (two doses of AstraZeneca-Oxford followed by one Pfizer booster).

Antibody levels in both groups were assessed using the reactivity index at four time points: P1: prior to the second dose (in either the CoronaVac or AstraZeneca-Oxford arm); P2: between 90 and 180 days after the second dose; P3: prior to the third Pfizer dose (also between 90 and 180 days after the second dose); P4: between 90 and 180 days after the third (Pfizer) dose, administered to both groups. These first four time points (P1-P4) were sufficient to evaluate humoral immunity. The fifth time point (P5), collected between 181 and 270 days after the Pfizer booster, was used to assess the longevity of cellular immunity.

**Fig. 2:**
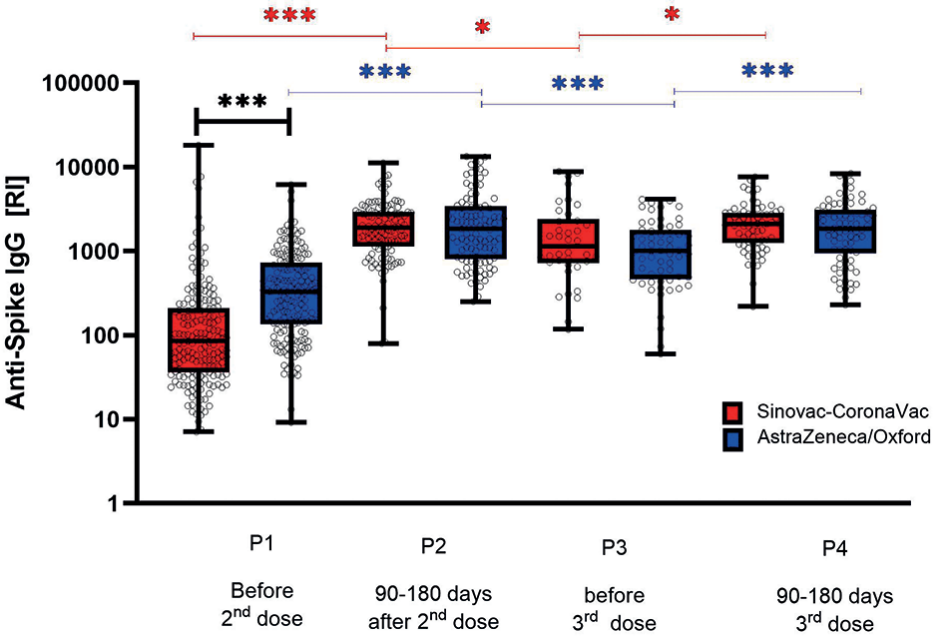
monitoring of anti-Spike antibody levels in the two coronavirus disease 19 (COVID-19) vaccine arms. The two vaccine arms comprised: the Sinovac-CoronaVac arm [two doses of Sinovac-CoronaVac followed by a booster dose with Pfizer (BNT162b2)] and the AstraZeneca-Oxford arm [two doses of AstraZeneca-Oxford followed by a booster dose with Pfizer]. Monitoring of total anti-Spike IgG levels was performed using an enzyme-linked immunosorbent assay (ELISA), and antibody responses were quantified by calculating the reactivity index (RI). The figure presents a comparison of the mean RI across four time points (P): P1) before the 2nd dose (whether in the Sinovac-CoronaVac or AstraZeneca-Oxford arm); P2) between 90- and 180-days after the 2nd dose; P3) before the 3rd dose (Pfizer); and P4) between 90- and 180-days after the 3rd dose, where Pfizer was used for immunization in both arms. Comparisons of mean RI values were conducted using the Mann-Whitney test. Statistical significance is indicated as follows: ^*^p < 0.05; ^**^p < 0.005; ^***^p < 0.0005.

Figure 2 compares anti-Spike antibody levels in participants after the first vaccine dose (prior to the second dose), followed by three subsequent time points in each vaccination arm. It is noteworthy that, within the national COVID-19 vaccination programme, the average interval between the first and second doses was approximately three months, while the interval between the second and third doses exceeded 180 days. From the first to the second time point, a significant increase in antibody levels was observed in both groups (p < 0.005). In the Sinovac-CoronaVac arm (shown in red, Fig. 2), the median antibody level increased by nearly 1 log unit, rising from below 100 at the first time point to above 1,000 at the second. In contrast, participants in the AstraZeneca-Oxford arm (shown in blue, Fig. 2) already exhibited median antibody levels above 10² at P1, which further increased to approximately 10³ at P2.

The third dose in the immunisation plan was made available six months after the second dose. When analysing antibody levels prior to the administration of the third dose - approximately one year after the second dose of the respective vaccine - a statistically significant reduction in antibody levels was observed in both arms when compared to the second time point. This decline was slightly more pronounced in the AstraZeneca-Oxford arm (p < 0.0005). Following the third dose, a statistically significant increase in antibody levels was detected (^*^or p < 0.05 for the Sinovac-CoronaVac arm and ^***^or p < 0.0005 for the AstraZeneca-Oxford arm), with antibody titres returning to levels comparable to those observed after the second dose.

**Fig. 3:**
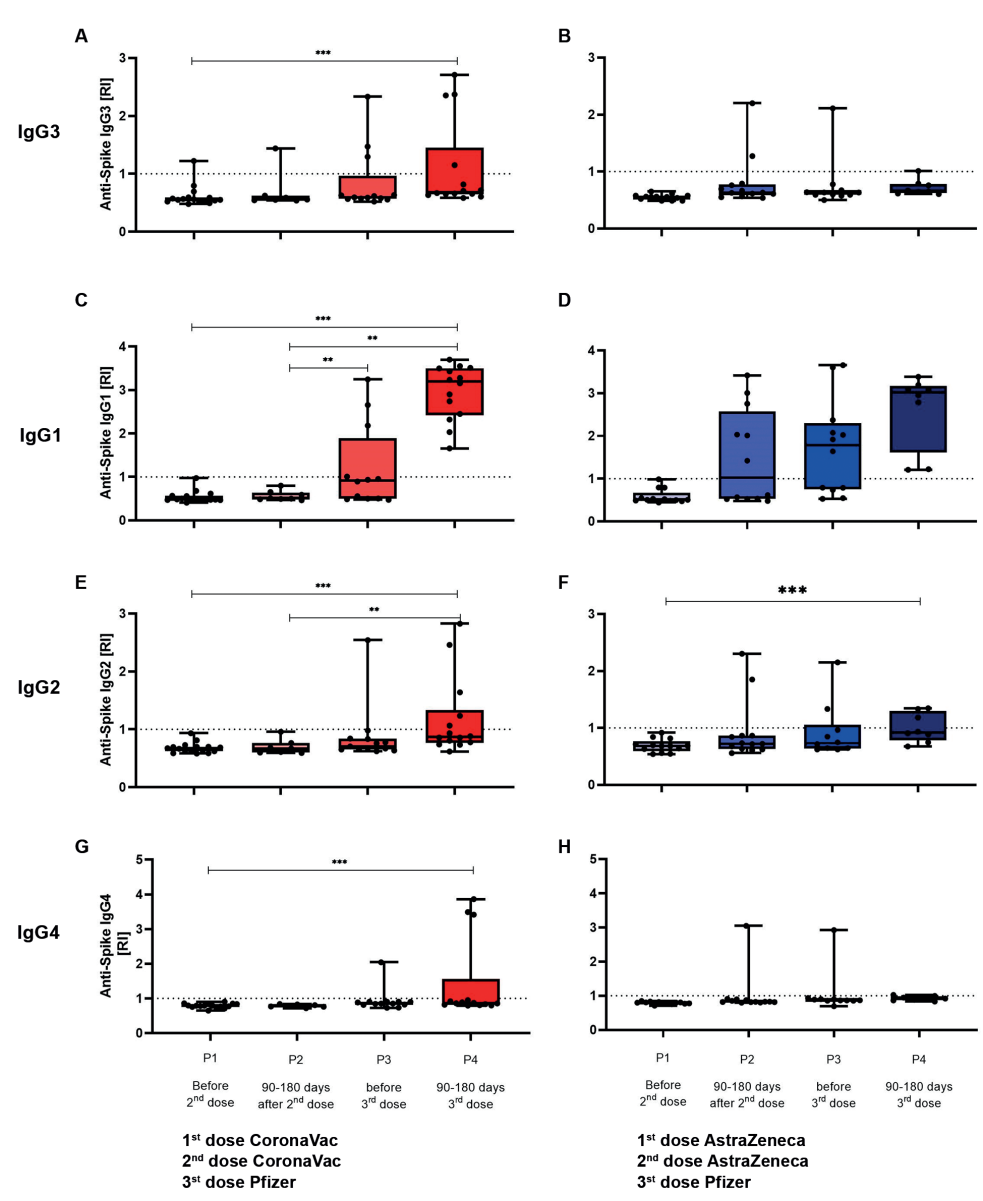
evaluation of IgG subclass responses. Anti-Spike IgG subclass levels were assessed using an enzyme-linked immunosorbent assay (ELISA), and responses were quantified by calculating the reactivity index (RI). A subset of 29 serum samples from individuals in both vaccine arms was monitored at four collection time points: P1) before the 2nd dose (whether in the Sinovac-CoronaVac or AstraZeneca-Oxford arm); P2) between 90- and 180-days after the 2nd dose; P3) before the 3rd dose (Pfizer); and P4) between 90- and 180-days after the 3rd dose Panel A represents data from the Sinovac-CoronaVac arm (N = 16); Panel B shows data from the AstraZeneca-Oxford arm (N = 13). Comparisons of mean RI values were conducted using the Mann-Whitney test. Statistical significance is indicated as follows: ^*^p < 0.05; ^**^p < 0.005; ^***^p < 0.0005.

*Comparison of anti-Spike IgG antibody isotypes (subclasses) in the two vaccine arms* - The dynamics of the IgG subclass response likely follow a sequential pattern based on the genomic organisation of the immunoglobulin heavy chain locus. The gamma 3 heavy chain gene (IgG3) is positioned immediately downstream of the mu and delta chains, making it the first to be expressed, followed by gamma 1 (IgG1), and subsequently gamma 2 (IgG2) and gamma 4 (IgG4), which are located further downstream. To better understand the subclass-specific IgG response, a subset of 29 serum samples was selected from a larger sample repository, each containing at least three paired samples per participant, analysed across four study time points (Fig. 3). In the Sinovac-CoronaVac arm, serum samples from 16 participants were tested at P1, with 8 retested at P2, 12 at P3, and 14 at P4. One participant (6.25%) had detectable IgG3 anti-Spike antibodies prior to the second dose at P1. At P2, 14.3% (1/7) remained IgG3-positive 90-180 days after the second dose, suggesting either a low but persistent response or individual variability. At P3, 23.1% (3/13) retained IgG3 positivity beyond 180 days, and at P4, the prevalence remained stable at 28.6% (4/14) following the Pfizer booster (third dose in the Sinovac-CoronaVac arm).

For IgG1, 33.3% (4/12) maintained anti-Spike antibodies at P3, more than 180 days after the second dose. This seroprevalence increased to 100% (14/14) at P4 following the Pfizer booster, reinforcing its role in sustained immunity. Regarding IgG2, none of the samples were positive at P1 or P2; however, 7.7% (1/13) showed detectable IgG2 anti-Spike antibodies at P3, increasing to 37.5% (5/14) at P4. For IgG4, no samples were positive at P1 or P2, while 7.7% (1/13) showed IgG4 reactivity at P3, increasing to 21.4% (3/14) at P4.

In the AstraZeneca-Oxford arm, samples from 13 participants were analysed at P1, with 12 tested at P2 and P3, and 8 at P4. Before the second dose, none of the 13 samples tested positive for IgG3 anti-Spike antibodies. However, at P2, 16.7% (2/12) were IgG3-positive, followed by 10% (1/10) at P3, and 12.5% (1/8) at P4. For IgG1, 50% (6/12) retained anti-Spike antibodies 90-180 days after the second dose (P2), a prevalence that remained stable at P3 (7/12), supporting the notion of a sustained immune response. Following the Pfizer booster (third dose), all tested participants (8/8) were IgG1-positive between 90 and 180 days post-booster at P4.

Regarding IgG2, none of the samples were positive before the second dose; however, at P2, 15.4% (2/13) showed detectable IgG2, with the same individuals remaining positive at P3 (20%, 2/10). At P4, IgG2 prevalence increased to 37.5% (3/8). For IgG4, none of the 13 samples were positive at P1, but 7.7% (1/13) were positive at P2, increasing to 9.1% (1/11) at P3 and 12.5% (1/8) at P4.

The low prevalence of IgG3 at time point P2 may reflect either a weak immune response or the transient nature of this subclass. In contrast, the IgG1 response at P2 may reflect a decline in seroprevalence in the Sinovac-CoronaVac arm, whereas it appears to be more sustained in the AstraZeneca-Oxford arm. Following the Pfizer booster, IgG1 seroprevalence remained stable, suggesting its role in long-term immunity. These findings align with the predicted hierarchy of subclass switching, where IgG1 and IgG3 predominate in early responses, while IgG2 and IgG4 emerge later, potentially indicating immune response maturation.

**Fig. 4:**
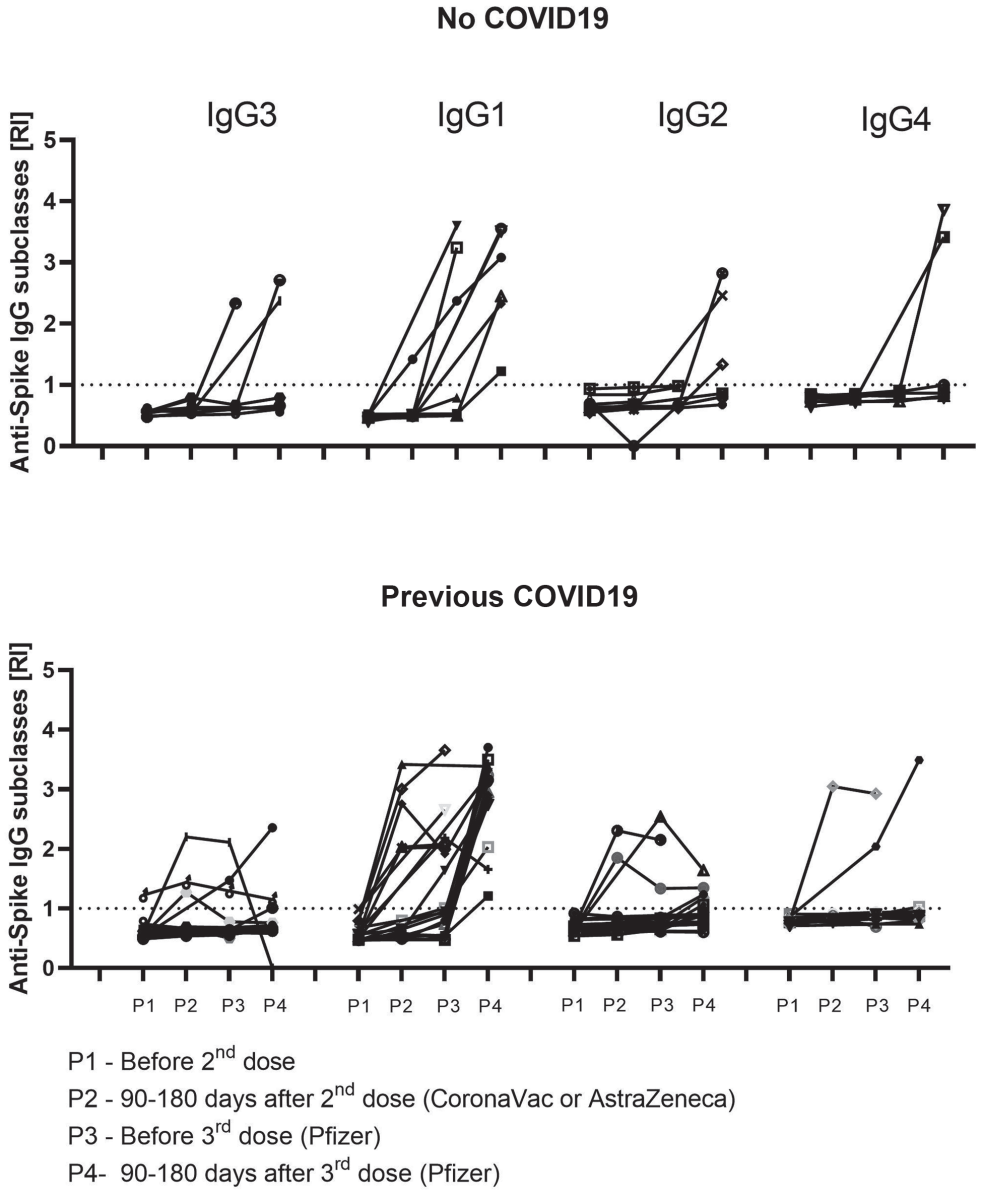
kinetic description of IgG subclass response in relation to previous coronavirus disease 2019 (COVID-19) infection. A subset of 29 serum samples from individuals in both vaccine arms was monitored at all collection time points (P). Anti-Spike IgG subclass levels were assessed using an enzyme-linked immunosorbent assay (ELISA), and responses were quantified using the reactivity index (RI). Panel A includes data from nine participants who had not experienced COVID-19 infection since the start of vaccination and throughout the study period. Panel B includes data from twenty participants who had confirmed COVID-19 in 2020. The four time points are as follows: P1) before the 2nd dose (whether in the Sinovac-CoronaVac or AstraZeneca-Oxford arm); P2) between 90- and 180-days after the 2nd dose; P3) before the 3rd dose (Pfizer); and P4) between 90- and 180-days after the 3rd dose.

To assess whether prior exposure to SARS-CoV-2 could influence IgG subclass switching, samples from both vaccination arms were pooled and categorised into two groups: (1) participants who reported prior exposure to SARS-CoV-2 without a confirmed COVID-19 diagnosis (N = 9), and (2) participants with no reported exposure (N = 20) (Fig. 4). For IgG3, none of the nine tested samples from participants without prior infection were positive at P1 or P2. However, at P3, 16.7% (1/6) maintained IgG3 anti-Spike antibodies approximately 12 months post-vaccination, when blood was collected immediately before the third dose. At P4, 33.3% (2/6) sustained IgG3 positivity 90-180 days after the Pfizer booster (Fig. 4A). Among those with prior COVID-19, IgG3 anti-Spike responses were more persistent: one of the 20 tested samples (5%) was positive at P1, increasing to 23.1% (3/13) at P2, 16.7% (3/18) at P3, and 17.6% (3/17) at P4 (Fig. 4B).

A similar pattern was observed for IgG1 post-P2. Among individuals without prior infection, 14.2% (1/7) were positive at P2, increasing to 50% (3/6) at P3, and 100% (6/6) at P4 (Fig. 4A). Among those previously infected with SARS-CoV-2, 38.4% (5/13) were IgG1-positive at P2, 44.4% (8/18) at P3, and 100% (16/16) at P4 (Fig. 4B). For IgG2 and IgG4, none of the nine samples from individuals without prior infection were positive at P1, P2, or P3. At P4, 50% (3/6) had sustained IgG2 responses, and 33.3% (2/6) sustained IgG4 responses (Fig. 4A). Among those reporting previous COVID-19, 14.3% (2/14) had IgG2 responses at P2, increasing to 15% (3/20) at P3 and 27.7% (5/18) at P4 (Fig. 4B). For IgG4, 7.1% (1/14) tested positive at P2, 10% (2/20) at P3, and 11.1% (2/18) at P4.

When comparing both groups, previous COVID-19 infection appears to be associated with a more sustained immune response. Although IgG1 exhibited comparable long-term responses between the groups, the seroprevalence of IgG3, IgG2, and IgG4 remained low between 90 and 180 days after the second vaccine dose (P2), particularly in the AstraZeneca-Oxford arm, and persisted at low levels beyond 180 days (P3). This suggests the presence of a residual humoral response in individuals previously exposed to SARS-CoV-2, which may contribute to prolonged immunity.

**Fig. 5:**
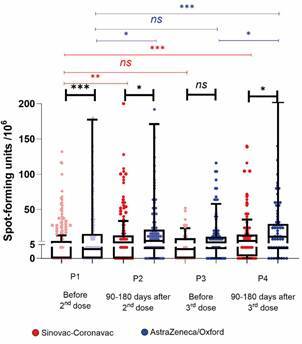
monitoring of IFNγ-producing cells in response to the Spike antigen in the two vaccine arms. The two vaccine arms consisted of: the Sinovac-CoronaVac arm [two doses of Sinovac-CoronaVac followed by a booster dose with Pfizer (BNT162b2)] and the AstraZeneca-Oxford arm [two doses of AstraZeneca-Oxford followed by a booster dose with Pfizer]. The number of interferon gamma (IFNγ)-producing cell colonies, or spot-forming units (SFU), in peripheral blood mononuclear cells (PBMCs) was monitored in response to stimulation with 253 peptides spanning the full length of the SARS-CoV-2 Spike protein (T-SPOT.COVID, Oxford Immunotec). The figure presents a comparison of the mean SFU counts at four time points (P): P1) before the 2nd dose (whether in the Sinovac-CoronaVac or AstraZeneca-Oxford arm); P2) between 90- and 180-days after the 2nd dose; P3) before the 3rd dose (Pfizer); and P4) between 90- and 180-days after the 3rd dose, where Pfizer was used for immunisation in both arms. Comparisons of mean SFU counts were performed using the Mann-Whitney test. Statistical significance is indicated as follows: ^*^p < 0.05; ^**^p < 0.005; ^***^p < 0.0005.

*Comparison of IFNγ-producing cell numbers in response to Spike antigen in the two vaccine arms* - Fig. 5 presents the cellular immune response assessed via IFNγ production in PBMCs using the ELISPOT assay, following the same sampling dynamics across four time points. The Sinovac-CoronaVac + Pfizer booster arm is depicted in red. From P1 to P2, there is a significant increase in the number of IFNγ-producing cells (p < 0.0005), which is maintained up to six months post-second dose, at P3. Following the third dose (P4), there is an increase in the mean number of IFNγ-producing cells compared to the first dose of Sinovac-CoronaVac (P1; p < 0.0005), and a trend towards a higher response than that observed immediately prior to the third dose (P3; p = 0.056). The AstraZeneca-Oxford + Pfizer booster arm is shown in blue and demonstrates a similar pattern. The average number of IFNγ-producing cells increases following the second dose of AstraZeneca-Oxford (P2; p < 0.05), with no evident decline over the six-month period leading to the third dose (P3). A significant increase is observed at P4 (after the third dose), when compared to both the first dose (P1) and the six-month interval preceding the booster (P3) (p < 0.05). When comparing the kinetics of cellular response between the two arms, it is evident that the AstraZeneca-Oxford regimen elicited a higher average number of IFNγ-producing cells than the Sinovac-CoronaVac regimen at three distinct time points: P1, P2, and P4.

**Fig. 6:**
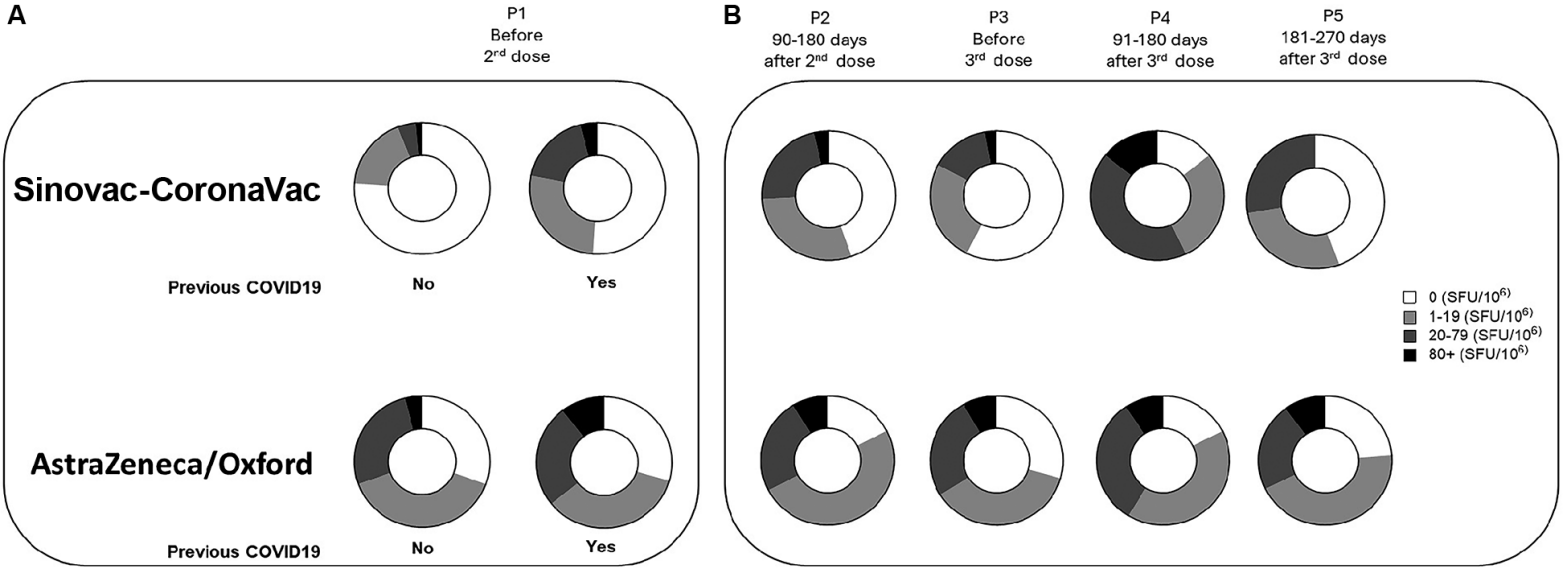
comparison of the frequencies of IFNγ-producing responders to vaccination across four time points in the two vaccine arms. The number of interferon-gamma (IFNγ)-producing cell colonies was categorised according to the number of spot-forming units (SFU) and distributed into the following response groups: non-responders = 0 SFU; low responders = 1-19 SFU; medium responders = 20-79 SFU; and high responders = ≥ 80 SFU (+ 80 SFU). Panel A presents a comparison of responder frequencies after the first dose of Sinovac-CoronaVac or AstraZeneca-Oxford, stratified by prior coronavirus disease 19 (COVID-19) infection status, based on the SFU categories defined above. Panel B shows the longitudinal follow-up of responder frequencies across the following time points: P2) between 90- and 180-days after the two doses of the primary immunisation in each arm; P3) six months later and before the 3rd dose (Pfizer); P4) between 90- and 180-days; and P5) between 181- and 270-days.

Figure 6 compares the number of IFNγ-producing cells categorised according to the number of spot-forming units (SFU), distributed into the following response categories: non-responders (0 SFU), low responders (1-19 SFU), medium responders (20-79 SFU), and high responders (≥ 80 SFU). In the Sinovac-CoronaVac arm, having a prior SARS-CoV-2 infection appeared to influence immune memory before administration of the second dose (P1). Referred to as hybrid immunity, slightly more than half of the individuals with previous COVID-19 still exhibited no detectable IFNγ-producing cells (0 SFU) in response to the Spike protein, classifying them as non-responders. In contrast, among participants who did not report prior infection, the frequency of non-responders was greater than three-quarters (Fig. 6A). In the AstraZeneca-Oxford arm, the distribution of response categories - ranging from 1-19 SFU to ≥ 80 SFU - was similar between individuals with and without a history of COVID-19, suggesting a more consistent induction of cellular immunity regardless of prior exposure.

In terms of follow-up (Fig. 6B), non-responders and individuals with 1-19 SFU in the Sinovac-CoronaVac arm represented the highest frequency after the second dose (P2). Six months later, prior to the third dose (P3), the frequency of non-responders returned to over 50%, while the proportions of low responders (1-19 SFU), medium responders (20-79 SFU), and high responders (≥ 80 SFU) declined accordingly. Following the Pfizer booster (P4), the frequencies of low, medium, and high responders increased considerably (Fig. 6B). However, between 90 and 180 days post-booster, the proportions of medium and high responders began to decrease. By 181-270 days after the third dose (P5), none of the participants remained in the high-responder category; nearly half were classified as non-responders, while the remainder were distributed between low and medium responder categories.

In the AstraZeneca-Oxford arm, the frequency of low responders comprised half of the participants following the second dose, followed by medium responders and non-responders, with the proportion of high responders remaining unchanged. Six months later (P3), prior to the Pfizer booster, there was a trend towards an increased frequency of non-responders, attributed to a decline in the proportion of low responders. The frequencies of medium and high responders remained stable. At P4, there was an increase in the frequencies of both low and medium responders, while the frequency of high responders remained unchanged. By 181-270 days post-booster (P5), the distribution of responder categories resembled that observed at P2 - 90 to 180 days after the second dose of AstraZeneca-Oxford.

## DISCUSSION

The development of COVID-19 vaccines progressed at an accelerated pace, with their application beginning in 2021 and proven efficacy against severe cases by 2022.^5^ ^,^ ^7^ ^,^ ^8^ ^,^ ^17^ ^,^ ^18^ The emergence of new variants necessitated booster doses and the adoption of heterologous vaccination regimens.^19^ ^,^ ^20^ In Brazil, as in other countries, the national COVID-19 vaccination programme employed a range of different vaccines.^11^ Combined analysis of humoral and cellular responses is crucial for predicting vaccine efficacy, and the incorporation of heterologous vaccination regimens has been shown to significantly extend the longevity of protection against COVID-19.^21^ ^,^ ^22^ ^,^ ^23^ Longer-term protection has become a subject of considerable international interest. To date, this study is the first to simultaneously compare the kinetics of binding antibody titres, IgG subclass profiles, and specific IFNγ responses to the SARS-CoV-2 Spike protein in a longitudinal framework.

T-cell responses appear to play an important role in reducing disease severity and conferring long-term protection. When comparing the immune responses elicited by the Sinovac-CoronaVac and Oxford-AstraZeneca vaccines, we observed that breakthrough infections occurred predominantly among individuals vaccinated with Sinovac-CoronaVac, and that this lower efficacy was associated with reduced antibody levels, particularly in those without prior.^12^ In the present study, anti-Spike IgG levels following the first dose of AstraZeneca-Oxford were higher compared to Sinovac-CoronaVac, which required two doses to reach the antibody titres observed after a single dose of AstraZeneca-Oxford, consistent with findings reported in other studies.^24^ ^,^ ^25^

There is evidence of a progressive decline in immunity over time, raising concerns about protection against new infections.^26^ Even with the observed decline in anti-Spike antibody levels prior to the third dose (six to eight months after the second dose), titres did not fall below those recorded after the first dose in either arm. Another important detail relevant to the management of future vaccination strategies is that the third Pfizer dose restored antibody levels to those observed following initial vaccination in each arm, consistent with findings from other studies.^27^ Furthermore, heterologous immunisation with Pfizer in both arms maintained anti-Spike IgG levels for more than 270 days and likely without significant decline over a 12-month period. This information is critical for informing decisions regarding extended intervals between doses and the scheduling of future booster vaccinations, as supported by findings in another study.^23^

Recently, a study demonstrated the production of IgG subclasses in two cohort studies following the administration of two doses of the SARS-CoV-2 mRNA vaccine.^28^ It is well established that IgG class switching is a genetic recombination event that is tightly regulated during the immune response. IgG3 is encoded by the Cγ3 region, which is the most upstream (5′) Cγ region in the locus, while the other IgG heavy chain genes - γ1, γ2, and γ4 - are located further downstream and encode IgG1, IgG2, and IgG4, respectively.^29^ With prolonged activation in the germinal centres of lymphoid organs, the constant region to which a B cell switches is modulated by cytokines and B cell activators at the transcriptional level of unrearranged heavy chain constant genes.^29^ ^,^ ^30^ Although this was a sample-based analysis, the anti-Spike IgG subclass profile revealed two noteworthy findings. Firstly, our results demonstrated that IgG1 predominated in both vaccine arms across all four time points, while IgG2 and IgG4 emerged in a subset of individuals over time. Similarly, Irrgang et al. reported IgG1 predominance shortly after administration of the initial two doses of the mRNA vaccine. However, IgG2 - and especially IgG4 - antibodies were observed to appear five to seven months after the second immunisation and following the third dose of the mRNA vaccine.^28^ The IgG2 and IgG4 subclasses are generally considered non-inflammatory, as they mediate effector functions associated with cellular immune responses.^29^ ^,^ ^31^ Nevertheless, caution is warranted in interpreting the role of IgG4, as it remains the least understood of the human IgG subclasses from both evolutionary and functional perspective.^32^ Therefore, the low prevalence of IgG2 and IgG4 observed among our participants may be attributable to the fact that they received only a single dose of the mRNA vaccine as a booster. According to Irrgang and colleagues, the emergence of IgG4 occurred predominantly after three doses of the Pfizer vaccine.^28^

Prior SARS-CoV-2 infections act as natural boosters, enhancing IgG levels reactive to the Spike protein.^25^ Our findings further elucidate the impact of prior exposure on the humoral immune response over time, particularly in the persistence of IgG3, IgG2, and IgG4 subclasses beyond 180 days post-vaccination. This observation is consistent with the study by Paula et al., which reported increased anti-Spike IgG levels in individuals vaccinated with CoronaVac and ChAdOx1-S who had previously been infected with SARS-CoV-2.^25^ Notably, IgG1 levels remained comparable between previously infected and naïve individuals, while the residual seroprevalence of IgG3, IgG2, and IgG4 suggests that natural infection functions as a priming event, thereby extending vaccine-induced immunity. Although prior infection initially confers an advantage, the administration of booster doses ultimately harmonises the humoral response across groups. This was similarly observed by Paula et al., in whose study a third Pfizer dose eliminated differences in IgG levels between previously infected and uninfected individuals.^25^ These findings underscore the complex interplay between natural infection and vaccination, reinforcing the critical role of booster doses, and their timing, in sustaining a robust and durable immune response.

Unlike humoral immunity, a progressive decline in cellular responses has been observed six months after the administration of two vaccine doses.^33^ In the Sinovac-CoronaVac arm, prior SARS-CoV-2 infection influenced the average number of IFNγ-producing cells; however, nearly half of the individuals were classified as non-responders six months after the second dose. The Pfizer booster dose reversed this trend, resulting in increased frequencies of low, medium, and high responders. Nevertheless, these frequencies declined again between 181- and 270-days post-booster. In the AstraZeneca-Oxford arm, the majority of participants were classified as low responders following the second dose, with relatively stable frequencies over time, except for an increase in non-responders observed prior to the administration of the booster dose. When comparing the immune response elicited by the two vaccine regimens, it became evident that the average number of IFNγ-producing cells was consistently higher in the AstraZeneca-Oxford arm compared to the Sinovac-CoronaVac arm. This kinetic analysis supports the prediction that the optimal interval for booster dose administration should be approximately six months, as indicated by other studies.^20^ ^,^ ^23^ ^,^ ^24^ ^,^ ^34^

Finally, our study investigated both humoral and cellular immune responses following primary immunisation in two vaccine arms and monitored these responses over a 20-month period (2021 to 2022). Our findings are consistent with previous studies indicating that humoral immunity, as measured by antibody levels, declines more slowly than cellular immunity.^35^ ^,^ ^36^ Although IFNγ-producing cells were still detectable in a substantial proportion of participants 180 days after the Pfizer booster, the frequency of non-responders was considerable, suggesting that both types of immunity wane over time, with cellular immunity appearing to decline more rapidly.

Our study has several limitations. Throughout the data collection period, we experienced sample losses at various time points, particularly at P3, P4, and P5. The study design focused on evaluating the absolute number of individuals from the DETECTCoV cohort who returned for successive follow-up collections. Another limitation was the absence of serological testing at time point P5. At this stage, the primary interest was to assess cellular immunity, given that a reduction in circulating IFNγ-producing cells had already been observed in one of the vaccine arms, while antibody levels remained elevated at P4. Additionally, it was not possible to perform subclass-specific serology in a larger number of samples or over a longer follow-up period, which may have limited our ability to detect more subtle variations in the humoral response.

In conclusion, our results demonstrate that although the initial anti-Spike IgG antibody response induced by Sinovac-CoronaVac was lower than that elicited by AstraZeneca-Oxford, the second dose equalised antibody levels between the two vaccine arms. The Pfizer booster dose proved effective in sustaining high antibody levels for over 270 days. However, cellular immunity, as assessed by IFNγ production, was less robust in the Sinovac-CoronaVac + Pfizer arm. A significant decline was observed between 90 and 180 days post-booster, with a lack of cellular response indicated by the absence of IFNγ-producing cells in nearly half of the individuals - classified as non-responders - six months after the booster. These findings underscore the need for differentiated booster strategies to ensure durable immunological protection. They also support the consideration of an annual booster schedule, akin to influenza vaccination, should COVID-19 transition into a seasonal disease.
