# ERRATUM: A 20-month longitudinal study to evaluate humoral and cellular immunity after COVID-19 vaccines

**DOI:** 10.1590/0074-02760240193er

**Published:** 2026-10-30

In the article **“A 20-month longitudinal study to evaluate humoral and cellular immunity after COVID-19 vaccines”**, DOI number: 10.1590/0074-02760240193, published in *Mem Inst Oswaldo Cruz*, Rio de Janeiro, Vol. 120: e240193, 2025, on page 1:

**Where it reads:**

Financial support: This study was supported by the Cooperation Agreement NU2GGH002174 between the ENSP, FIOCRUZ, FIOTEC and CDC (USA). Scholarships were granted by the FAPEAM through the POSGRAD/FAPEAM program to WBSS and BBS; PJL received project support through the PCTI-EMERGESAÚDE/AM - CHAMADA I - ÁREAS PRIORITÁRIAS/FAPEAM; PAN received project support through the PCTI-EMERGESAÚDE/AM - CHAMADA II - ÁREAS PRIORITÁRIAS/FAPEAM; scholarships for BBS, LPV and LDN were provided through the PCTI-EMERGESAÚDE/AM - CHAMADA II - ÁREAS PRIORITÁRIAS program, supported by FAPEAM. This work was also supported by the CAPES (Funding Code 001).

**It should read:**

Financial support: This study was supported by the Cooperation Agreement NU2GGH002174 between the ENSP, FIOCRUZ, FIOTEC and CDC (USA), and by the Coronavirus Aid, Relief, and Economic Security Act (CARES) through the Centers for Disease Control and Prevention (CDC) under the terms of NU2GGH002174 / 0900f3eb81ee4293. Scholarships were granted by the FAPEAM through the POSGRAD/FAPEAM program to WBSS and BBS; PJL received project support through the PCTI-EMERGESAÚDE/AM - CHAMADA I - ÁREAS PRIORITÁRIAS/FAPEAM; PAN received project support through the PCTI-EMERGESAÚDE/AM - CHAMADA II - ÁREAS PRIORITÁRIAS/FAPEAM; scholarships for BBS, LPV and LDN were provided through the PCTI-EMERGESAÚDE/AM - CHAMADA II - ÁREAS PRIORITÁRIAS program, supported by FAPEAM. This work was also supported by the CAPES (Funding Code 001).


Adeilton Brandão
*Editor-in-Chief*
Memórias do Instituto Oswaldo Cruz


